# The Circadian Clock, the Immune System, and Viral Infections: The Intricate Relationship Between Biological Time and Host-Virus Interaction

**DOI:** 10.3390/pathogens9020083

**Published:** 2020-01-27

**Authors:** Gianluigi Mazzoccoli, Manlio Vinciguerra, Annalucia Carbone, Angela Relógio

**Affiliations:** 1 Department of Medical Sciences and Chronobiology Laboratory, Fondazione IRCCS “Casa Sollievo della Sofferenza”, 71013 San Giovanni Rotondo (FG), Italy; annalucia.carbone@gmail.com; 2 Epigenetics, Metabolism and Aging (EMA) research group, International Clinical Research Center, St. Anne’s University Hospital, 656 91 Brno, Czech Republic; manlio.vinciguerra@fnusa.cz; 3 Institute for Theoretical Biology (ITB), Charité—Universitätsmedizin Berlin, corporate member of Freie Universität Berlin, Humboldt-Universität zu Berlin and Berlin Institute of Health, 10117 Berlin, Germany; 4 Medical Department of Hematology, Oncology, and Tumor Immunology, and Molekulares Krebsforschungszentrum (MKFZ), Charité—Universitätsmedizin Berlin, corporate member of Freie Universität Berlin, Humboldt-Universität zu Berlin and Berlin Institute of Health, 10117 Berlin, Germany

**Keywords:** clock, circadian rhythms, virus, host, immune system

## Abstract

Living beings spend their lives and carry out their daily activities interacting with environmental situations that present space-time variations and that involve contact with other life forms, which may behave as commensals or as invaders and/or parasites. The characteristics of the environment, as well as the processes that support the maintenance of life and that characterize the execution of activities of daily life generally present periodic variations, which are mostly synchronized with the light–dark cycle determined by Earth’s rotation on its axis. These rhythms with 24-h periodicity, defined as circadian, influence events linked to the interaction between hosts and hosted microorganisms and can dramatically determine the outcome of this interplay. As for the various pathological conditions resulting from host–microorganism interactions, a particularly interesting scenario concerns infections by viruses. When a viral agent enters the body, it alters the biological processes of the infected cells in order to favour its replication and to spread to various tissues. Though our knowledge concerning the mutual influence between the biological clock and viruses is still limited, recent studies start to unravel interesting aspects of the clock–virus molecular interplay. Three different aspects of this interplay are addressed in this mini-review and include the circadian regulation of both innate and adaptive immune systems, the impact of the biological clock on viral infection itself, and finally the putative perturbations that the virus may confer to the clock leading to its deregulation.

## 1. Introduction

Rotation of the planet Earth around its axis generates rhythmic day/night alternations with a period of approximately 24 h (circadian). The term circadian originates from the Latin *circa dies*, “approximately one day”, more or less the time length of day/night or light/dark cycles. Circadian rhythms, generated by the endogenous circadian clock, allow living beings to anticipate environmental daily changes and provide an evolutionary advantage that favours survival despite strong selective pressure. The circadian clock is evolutionary conserved and thought to be roughly 2.5 billion years old, tracing back to cyanobacteria, which started releasing vast amounts of oxygen during the Great Oxidation Event, prior (700 million years) to the divergence of plants, animals, and fungal lineages [1,2,3].

The biological clock is a molecular network which generates oscillations in gene and protein expression that control cellular functioning and allows for the timely separation of biological processes that cannot or should not coexist. These include redox reactions and DNA replication [4], switches in metabolic pathways [5,6,7], as well as behavioural and physiological rhythms [8]. The endogenously driven rhythms are synchronized by external cues, such as environmental light and temperature, and feeding times [4,7,9] and are resilient to temperature fluctuations within the physiological range [10,11].

### 1.1. The Circadian Clock Circuitry

In mammals, the system driving the generation of internal timing and ultimately the regulation of physiology and behaviour is formed by a hierarchical network of biological oscillators with two hubs in the hypothalamic suprachiasmatic nuclei (SCN) [12]. The SCN clock (main pacemaker) is necessary for the maintenance of 24-h body rhythms [13,14] and can restore [15] or de novo establish circadian rhythms in gene expression when transplanted in genetically arrhythmic mice [16,17]. The circadian circuitry encompasses molecular clocks of the main pacemaker and additional cellular clocks in peripheral tissues [18,19,20]. These are driven by the SCN through neural and humoral outputs [21] and, together with the main pacemaker, manage circadian timing of physiological processes and behaviour [2,22]. One of the main external synchronizers (or zeitgeber) is light, a strong central pacemaker-tuning cue [23]. Light information is perceived by retinal ganglion cells expressing the photopigment melanopsin and passed on to the SCN through retino-hypothalamic tract fibres [24,25,26,27] and to neighbouring and downstream oscillators via neurotransmitters such as glutamate, PACAP, VIP, GRP [25,28,29], VP [30,31], and GABA [32]. Even though the oscillators in the SCN are capable of autonomous ticking, the oscillations become progressively dampened in peripheral oscillators in the absence of SCN-synchronizing signals [33].

### 1.2. The Molecular Mechanisms of Biological Ticking

The molecular clockworks generate rhythmic oscillations through “transcription-translation feedback loops” (TTFL), i.e., core-clock transcription factors positively turn on the expression of genes encoding circadian proteins that subsequently inhibit transcription via negative-feedback loops, with a time delay indispensable for the proper functioning of the biological clock [34]. The TTFL is manoeuvred by several core-clock mRNAs and proteins, including activators (BMAL1, CLOCK, RORα, RORβ, and RORγ) and repressors (PER1, PER2, PER3, CRY1, CRY2, REV-ERBα, and REV-ERBβ), kinases (CKIα, CKIδ, and CKIε), and phosphatases (PP1 and PP5) that control protein stability and subcellular localization. CLOCK (or its paralogue NPAS2) and BMAL1 (alias ARNTL), as basic helix-loop-helix-PAS (bHLH–PAS) transcription factors [34] heterodimerize and bind to E-boxes in the promoter regions of the genes encoding the period (PER1, PER2, and PER3) and cryptochrome (CRY1 and CRY2) proteins, which upon heterodimerization in the cytoplasm translocate back into the nucleus and repress CLOCK:BMAL1-mediated transcription. Then, ubiquitination-dependent degradation of PER/CRY complexes takes place and a new round of CLOCK:BMAL1-mediated transcription restarts [34].

In addition, the nuclear receptors REV-ERBα and REV-ERBβ compete with RORα, RORβ, and RORγ at ROR-binding elements (ROREs) in the promoter region of *BMAL1* and fine-tune its transcription [34]. The CLOCK:BMAL1 heterodimer activates another auxiliary loop operated by the PAR-bZip transcription factors D-Box Binding PAR BZIP (DBP), Thyrotrophic Embryonic Factor (TEF), and Hepatic Leukemia Factor (HLF); bind to D-box-containing sites within the promoter region of the transcriptional repressor NFIL3 (alias E4BP4); and prompts numerous downstream transcriptional events [34].

### 1.3. Viruses and Circadian Clock Circuits

Synchronization with the external environment and anticipation of predictable environmental changes confer competitive advantages to living beings, especially in the wild. Accordingly, in humans experiencing dysregulation of circadian timing, for example, due to shift work or social jet lag, there is increased incidence of various degenerative, inflammatory, metabolic, and neoplastic diseases [35,36]. Besides human health and wellbeing maintenance, a detailed knowledge of the molecular mechanisms operating biological clocks has implications also for the efficacy of treatment strategies for pathological states.

**Figure 1:**
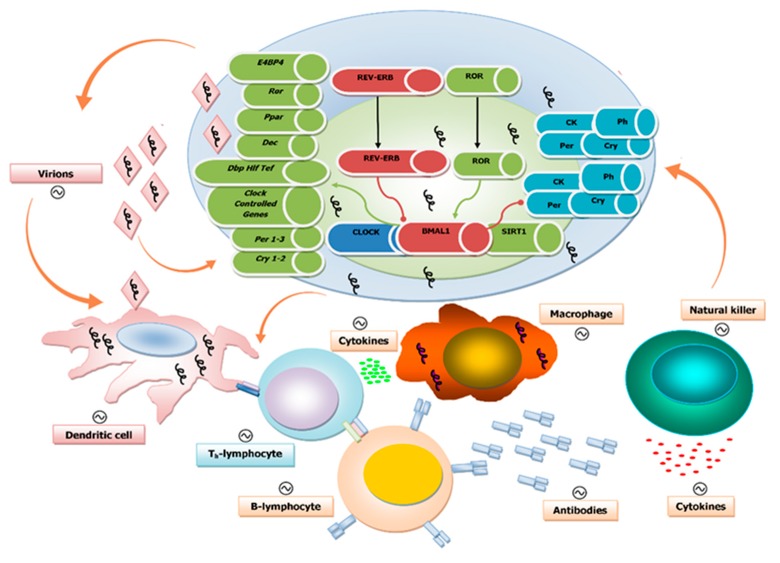
Schematic illustration of the interplay between the biological clock, virus replication, and the immune system at the cellular level: The components of the molecular clockwork are depicted within the cell, with green arrows indicating activation and red arrows indicating inhibition. The presence of viral genomes is indicated by squiggles. Below the cell are elements of the immune response. The orange arrows indicate interactions among virions, biological clocks, and immune competent cells. The interaction of virions and their nucleic acid core with these two players impacts viral replication and rhythmic patterns of host–hosted molecular trade off. The immune system with its innate and adaptive arms provides shielding against viral infections with a number of molecular factors and effectors, such as dendritic cells, T and B cells, macrophages, and natural killer cells. These are engaged to hold up and hinder virus replication and dissemination through the secretion of cytokines and the production of specific antibodies. The components and, ultimately, the complex function of the immune system are rhythmically driven by the biological clock and, in turn, influence the function of the molecular clockwork. Viral particles impact the interplay between immune and circadian systems.

Mounting evidence shows that time-of-day-related treatment schedules and chrono-modulated drug delivery significantly affect both effectiveness and side effects of pharmacological therapy [37,38,39]. Diseases caused by viruses, rickettsiae, bacteria, fungi, or parasites continue to represent a primary cause of morbidity. Infections by viruses rework the biological processes of infected cells to facilitate replication and spread, and thus the molecular interplay between the mechanisms of the biological clock, immune system, and virus infection can influence disease outcomes (Figure 1).

### 1.4. Circadian Regulation of Both Innate and Adaptive Immune Systems

The circadian regulation of the immune system response allows for its time-dependent success against different pathogens in a rhythmic fashion [40,41,42,43,44]. Immune cells of the innate and adaptive components of the immune system have molecular clockworks managing their rhythmic processes on a 24-h timescale [45,46,47,48,49], such as lymphocyte migration through lymph nodes and lymph in mice [50].

The rhythmic function of the immune system influences the appearance of pathological conditions, for instance, inflammatory lung disease and asthma, which show circadian fluctuations in symptoms and occurrence. In a recent study using both acute and chronic models of virus-induced airway disease (based on the Sendai virus (SeV)), disturbance of the 24-h periodicity negatively affected development, progression, and exacerbation of asthma [51]. In this study, *BMAL*1 silencing lead to more pronounced asthma-typical airway changes, which suggests a role for *BMAL1* in the regulation of lung-specific antiviral responses and the subsequent development of asthmatic symptoms. This study reinforces the previous findings from Majumdar et al., who reported a role for BMAL1 as a regulator of innate immunity and showed that BMAL1-deficent cells are more susceptible to infection of the RNA viruses RSV (respiratory syncytial virus) and PIV3 (parainfluenza virus type 3) [52].

The efficacy of vaccination is also thought to be under circadian control as suggested by results obtained in mice and human studies. Silver et al. have shown in immunized mice that vaccination at the time of higher TLR9 (Toll-like receptor 9) expression led to enhanced immune response, likely due to the circadian expression pattern of TLR9 [44]. Interestingly, in the same study, *PER2*-deficient mice did not show the observed time variation of immune response. Likewise, the antibody response to viral challenge in humans was also reported to be time dependent: immunization with hepatitis A and influenza virus vaccines in the morning led to higher antibody response as compared to afternoon vaccination [53,54,55].

### 1.5. Influence of the Biological Clock on Virus Replication Cycle and Disease

Cellular and tissue functions exhibit rhythmic fluctuations over the course of 24 h that impact the susceptibility to or progression of the viral infection. Several core-clock elements have been reported to modulate viral infection. BMAL1 and PER2 are regulators of antiviral immunity, as described in the previous section, and additional roles for PER1 and REV-ERBα, as well as melatonin have been highlighted, as described in the following. The seasonal variation of BMAL1 expression, reaching its lowest values in winter, seems to contribute to the high occurrence of respiratory viral diseases in this season since low *BMAL1* expression enhances viral disease [56,57]. An effect of the circadian clock in viral infection was also reported for wild-type mice infected with murine herpesvirus 4 at different times of the day. The mice were kept under controlled temperature and lighting environment (12-h light/12-h darkness), and the levels of infection and spread of the virus were evaluated [58]. In mice infected at the beginning of the day, corresponding to the beginning of the resting phase of these nocturnal animals, the viral replication was increased tenfold compared to the values measured in mice infected during the night period, corresponding to the activity phase. In mice lacking *BMAL1*, the levels of virus replication were independent of the time of day at which the viral infection was performed [58]. The effect of time of infection on virus replication was independent of the immune system, as also confirmed on single cell cultures (embryonic fibroblasts), in which several time-dependent processes (intracellular trafficking, biosynthetic processes, protein synthesis, and chromatin assembly) were involved. The obliteration of cellular circadian rhythms increased the replication of herpes and influenza A viruses, which lost rhythmic fluctuation and remained persistently high [58]. The time of day of herpes simplex virus type 2 (HSV-2) infection impacted the outcome of the pathology in wild-type mice kept under 12-h light and 12-h darkness (LD 12:12), probably due to the circadian rhythmic expression of the HSV-2 receptor in the skin Nectin1. This effect was confirmed in immunodeficient RAG2-knockout mice [59].

Moreover, the core-clock gene *PER1* was identified as a negative regulator of HIV-1 transcription and its expression inversely correlated with viral loads, likely in relation to different latencies of the virus in resting CD4+ T-cells [60].

Liver infection caused by hepatitis B and C viruses was also reported to be under circadian control. REV-ERBα plays an indirect role in Hepatitis C Virus (HCV) infection by negatively regulating miR122 [61], a microRNA known for its essential role in HCV replication [62]. In addition, PER2 overexpression decreased HCV RNA replication in permissive hepatocyte-derived cells. Interestingly PER2 can influence interferon signalling, which is a known player in regulating both the innate and the adaptive antiviral response and viral replication [46]. Enhanced expression of interferon was also observed as a result of melatonin administration in a mouse model of VEE (Venezuelan equine encephalomyelitis). Melatonin is a well-known component of the circadian system; this hormone is produced with 24-h rhythmicity by the pineal gland and has been associated with improved response against viruses. This study showed that melatonin administration results in decreased mortality upon VEE infection [63]. Melatonin was successfully applied against the respiratory syncytial virus (RSV), a pathogen causing severe disease in children, resulting in increased survival of newborn children with RSV-related respiratory sepsis [64]. In addition, the administration of melatonin in the treatment of individuals who are infected with the Ebola virus was reported to reduce several pathological changes caused by the virus to the host, and thus melatonin seems to be an interesting nontoxic candidate for future studies on antagonists of viral infections [65].

Another important interaction involves BMAL1 and the HSV transcriptional machinery. BMAL1 is able to associate with the regulatory protein ICP0 [66]; it forms heterodimers with the histone acetyltransferase CLOCK and localizes at ND10 nuclear bodies. Viral proteins stabilize the heterodimer and enzymatically activate CLOCK, which associates with the transcriptional complex (ICP4, ICP27, ICP22, and TFIID), linking the replicative cycle of the virus to the molecular clockwork [67].

### 1.6. Disruption of the Circadian Clock by Viruses

Viral infection may also alter circadian gene expression as reported in different studies. Simian immunodeficiency virus (SIV) infection in monkeys caused changes in amplitude and values of body temperature and locomotor activity circadian rhythmicity after the acute retroviral syndrome stage. These changes were not linked to modifications detected in the acute febrile response triggered by virus inoculation. On the other hand, hypothalamic microglia infiltration and macrophage accumulation were shown to occur in animals sacrificed upon appearance of strong circadian anomalies, hinting at analogously significant physiological and psycho-cognitive consequences in human subjects with HIV infection [68]. Moreover, in vitro experiments performed in a cell line (Bel-7404-HBx) stably transfected with the hepatitis B virus X protein (HBx, involved in hepatocellular carcinogenesis) showed alterations in the mRNA expression levels for several core-clock genes, with upregulation of *CLOCK*, *PER1*, and *PER2* and downregulation of *BMAL1*, *PER3*, *CRY1*, *CRY2*, and *CKIɛ* [69].

### 1.7. The Biological Clock and Influenza Virus Infection

A worldwide problem is represented by seasonal epidemics of influenza. Influenza is a disease with global impact that causes enormous morbidity and mortality on an annual basis. The severity of the infection depends on both the virus strain and a number of host factors, primarily age and the presence of comorbid conditions. The mortality and usage of healthcare resources associated with influenza is focused in the elderly and in those with a coexisting disease such as chronic obstructive pulmonary disease (COPD). In patients with COPD, the morbidity and mortality caused by an influenza virus infection are considerably greater.

In a COPD/emphysema mouse model, influenza A virus (IAV) infection reworked lung clock gene expression and decreased the amplitude in the rhythms of locomotor activity. This effect was more prominent in C57BL/6J mice that were chronically exposed to cigarette smoke and was accompanied by decreased body weight and augmented mortality; likewise, *Bmal1* knockout mice infected with IAV showed amplified lung inflammatory and pro-fibrotic responses.

Furthermore, the time of day of virus infection crucially impacts the outcome of IAV and this effect is observed under different types of light–dark cycles (12-h light/12-h darkness versus constant darkness), as well as in genetic circadian disruption models. The time of infection impacted late-viral clearance and IAV survival in *Bmal1^fl/fl Ercre+^* mice, and this time-of-day observed variation was abolished in *Bmal1^−/−^* mice. The rhythmic fluctuation of IAV-dependent effects was driven by inflammatory changes in the lung favouring better survival and related to a time point with high proportion of natural killer cells and of natural killer T lymphocytes and small percentages of inflammatory monocytes [70]. Accordingly, experiments performed in mice with targeted *Bmal1* deletion in pulmonary airway epithelial cells (AECs), cellular elements that vitally shape the host/environment boundary, induced alterations in neutrophil infiltration, biomechanical function, and responses to influenza infection in the lungs. Analysis of the 24-h time-resolved RNA sequencing performed on laser-captured AECs indicates that extensive transcriptomic changes in circadian enriched pathways are linked to lipid metabolism, xenobiotic detoxification, extracellular matrix, and chemokine signalling [71].

The *Bmal1* knockout findings suggest that proper functioning of the molecular clockwork is necessary for the immune response towards viral infections and the subsequent disease [72].

### 1.8. Epigenetic Mechanisms Underlying the Interaction Between Viral Infection and the Circadian Clock Machinery

Epigenetic regulatory mechanism, which includes DNA methylation and histone posttranslational modifications (PTM), leads to chromatin remodelling and regulated gene expression. Chromatin remodelling represents a critical process through which inputs, such as light or food, are transduced by the cell to generate permissive and silencing histone modifications to influence gene transcription and ultimately signalling pathway activity. CLOCK-BMAL1-dependent activation of clock-controlled genes is connected to circadian changes in histone PTM at their own promoters. Numerous chromatin remodelers, such as the deacetylases Sirtuin 1 (SIRT1, a NAD+-dependent histone deacetylase) and histone deacetylase 3 (HDAC3), are recruited to the promoter region of the clock-controlled genes in a circadian manner [34,73]. Importantly, the core element of the clockwork machinery, the transcription factor CLOCK, displays histone acetyltransferase activity as well. In the absence of these chromatin modifiers, the rhythmic expression of the clock-controlled genes is abrogated [73]. One of the most common epigenetic modifications is the methylation of DNA (addition of a methyl group to the cytosine residue in CpG dinucleotides mediated by the family of DNA methyltransferase enzymes DNMT1, DNMT3a, and DNMT3b). Overall DNA methylation with focal hypermethylation on the promoter of tumour suppressor genes, causing transcriptional silencing, is a mechanism of carcinogenesis [74,75,76]. These epigenetic mechanisms are critical for the functioning of the biological clock [73] and are disrupted by viral infection [74,75,76].

Previous studies report the existence of an interplay between the molecular clockwork, epigenetic modifications, and hepatitis C virus (HCV) in the liver, which affects ~3% of the population worldwide. Infection with HCV is considered one of the main risk factors for hepatocellular carcinoma (HCC) [77]. HCV is clustered into six main genotypes (in turn divided into various subtypes) [78]. Abundant evidence has demonstrated that HCV-related pathogenesis and its impact on liver disease is dependent on the genotype and that genetic and epigenetic mechanisms underlay viral interaction with cell processes and the molecular clockwork [79].

In the in vitro model of Huh-7 cells transfected with HCV core protein of different genotypes (1b, 2a, 3a, 4h, and 5a) [80], cellular DNMT1 and DNMT3b protein levels were increased in genotype 1b or 3a HCV core-expressing cells as compared to control cells [75]. Changes in the balance of DNA methylation mediated by DNMTs has been directly linked to HCV-dependent increase in hepatocarcinogenesis and in particular in the onset of hepatocellular carcinoma (HCC), hallmarked by global DNA hypomethylation and local hypermethylation on the promoter of tumour suppressor genes [74]. A recent meta-analysis showed that reduced E-cadherin (CDH1) expression is a predictor of poor prognosis in HCC patients [81]. CDH1 promoter is massively hypermethylated in genotype 1b HCV core protein-positive Huh-7 cells, in turn leading to reduced levels of CDH1 protein and increased levels of SIRT1 [76]. HCV-dependent alterations in SIRT1 function/activity have been shown by several studies performed in different cellular models [82,83,84,85,86]. In turn, SIRT1 is a well-established regulator of the circadian oscillation of clock-controlled genes in the liver, controlling fat metabolism, cell proliferation, and regeneration [87,88]. HCV has been shown to affect directly the circadian clock machinery. In Huh-7 cells expressing the HCV core protein genotype 1b, but not 3a, protein levels of PER2 and CRY2 were found decreased [79]. Reciprocally, overexpression of PER2 led to a consistent decrease in the levels of HCV RNA undergoing replication [79]. Furthermore, in liver biopsies from HCV genotype 1b-infected patients, PER2 was almost exclusively localized to the nucleus, which is suggestive of an auto-inhibitory transcriptional feedback loop [79]. More recently, it was shown that the core-clock components BMAL1 and REV-ERBα act upon more steps in the HCV life cycle, including the entry of viral particles into hepatocytes and the replication of the viral RNA [89]. In fact, pharmacological activation of REV-ERB inhibited HCV entry, and genetic knockout of *Bmal1* or overexpression/pharmacological activation of REV-ERB was able to counteract HCV replication by modulating the circadian expression of the liver-specific microRNA miR-122 with consequent derangements of signalling pathways involved in lipid metabolism [89]. A better understanding of circadian modulation of HCV life cycle and dynamic/rhythmic patterns of host–hosted interplay might lead to increased efficacy of therapies for HCV infection-related diseases.

## 2. Conclusions

Infectious agents exploit infected organisms in order to survive, multiply, settle, and eventually spoil. While some infections may remain asymptomatic or scarcely manifest, others are treatment resistant and have a grim prognosis. The immune system efficiently shields the body against infectious agents but eventually may fail, with harsh outcomes for the infected organism. Viruses are intracellular parasites hinging on the host cellular structures and cellular processes to replicate, survive, and disseminate. The biological clock drives circadian rhythmicity of cellular processes and immune functions, thus shaping the host response to viral infections and affecting the patterns of host–pathogen interaction [90]. Similarly to other infectious diseases, viral infections impact the circadian clock circuitry of the infected organism that, in turn, interacts with the molecular components of the infecting pathogens, pointing to a role of timing in this complex interplay and to a possible advantage of applying chrono-modulated antiviral therapeutic strategies. The contemporary chronotherapy employed or proposed in metabolic, immune, and neoplastic diseases could better address the outline of host–pathogen interaction, advancing the efficacy of antiviral therapy and improving the outcome of treatment of viral infections.

Even though much has been investigated in the circadian clock/immune system field in recent years, several open and outstanding questions remain to be addressed. Are different branches of the immune system differentially regulated by circadian rhythms? If autonomous ticking is dampened in peripheral tissues, do peripheral immune cells alter their rhythms/responses relative to immune cells in the thymus and lymph nodes? Do responses to vaccines that need boosters show different time-dependent patterns? Would different adjuvants boost vaccine responses in a time-of-day-dependent manner? Does deregulation of circadian rhythms correlate with reactivation of viruses known to cause latent infections (herpes simplex virus, varicella zoster virus, Epstein–Barr virus, human cytomegalovirus, human herpesvirus 6, human herpesvirus 7, Kaposi’s sarcoma-associated herpesvirus, JC virus, BK virus, parvovirus, and adenovirus)? Do hibernating animal species mount different antiviral responses during hibernation? These are extremely interesting topics for future research, and the answers to the questions above will help pave new directions in the study of circadian–immune network interactions in the context of viral infections.
